# Supplementary material for: The Construction and Exploration of a Comprehensive MicroRNA Centered Regulatory Network in Foxtail Millet (Setaria italica L.)
Source: Front Plant Sci. 2022 May 6;13:848474. doi: 10.3389/fpls.2022.848474 (PMC9121102; doi:10.3389/fpls.2022.848474)
Supplement: Supplementary file 3 [file Data_Sheet_3.zip › Sit-miRN2548_Seita.7G078300.1_2148_TPlot.pdf]

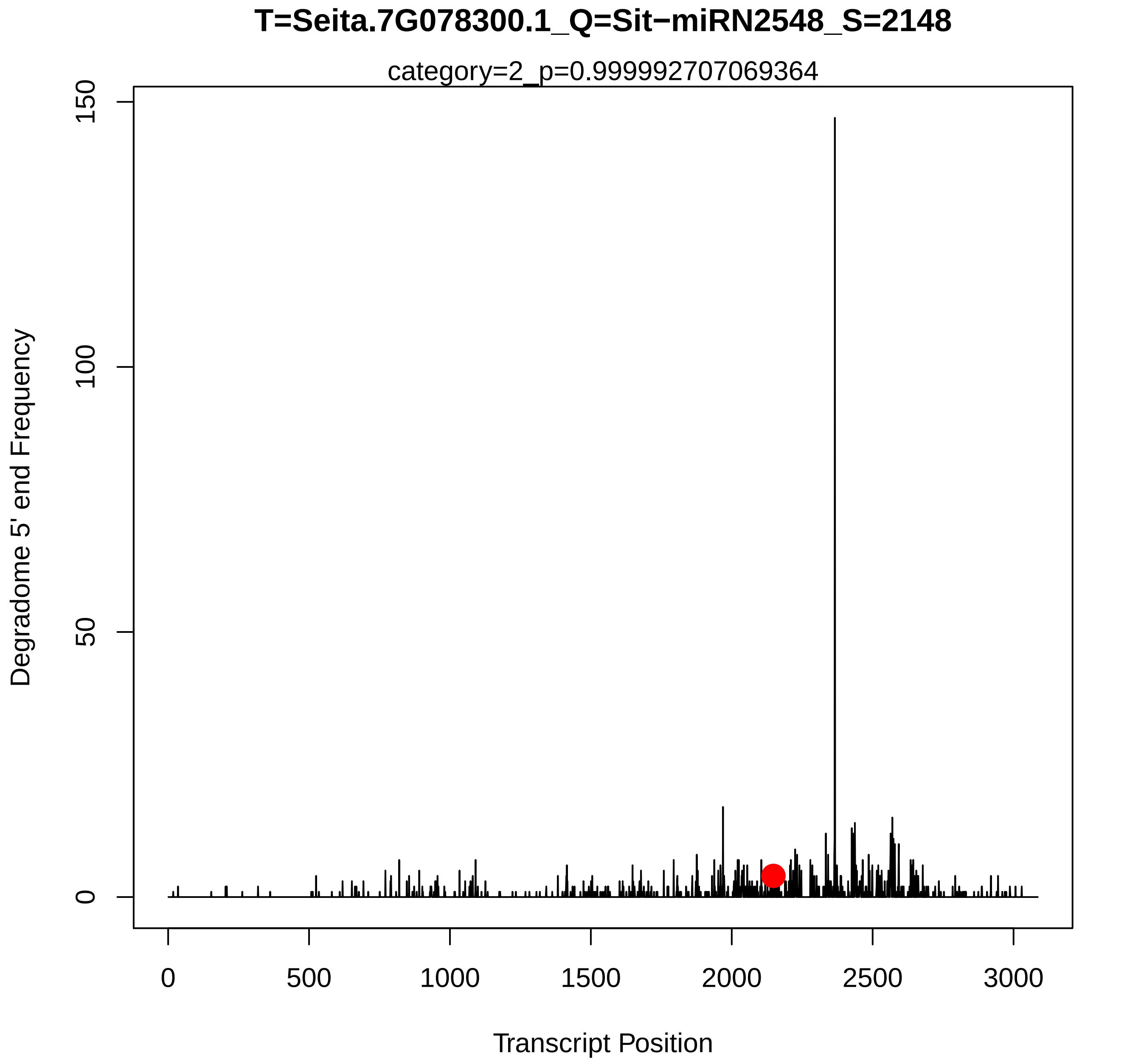

T=Seita.7G078300.1_Q=Sit−miRN2548_S=2148
category=2_p=0.999992707069364
Degradome 5' end Frequency
0
50
100
150
0
500
1000
1500
2000
2500
3000
Transcript Position
